# Holiday Camp Schools

**Published:** 1920-12-04

**Authors:** 


### THE PUBLIC WELL-BEING.

### Holiday Camp Schools.

### A VALUABLE EXPERIMENT DESCRIBED.

In our comments last week on the subject of Open-air Schools dealt with in the Report of the Chief Medical Officer of the Board of Education we promised to publish the experiences officially set forth by Dr. Hamer, the County School Medical Officer for London, with regard to holiday camps and schools. Dr. Hamer speaks with authority, and his account should be of great interest and value to all educational bodies throughout the kingdom. Although, he says, the amount of provision necessary for continuous open-air education of debilitated, anæmic, and tuberculous children is pretty well established, the needs of London in the matter of holiday or camp schools are difficult to estimate fully and accurately because of the varying demands at various times, the different standards that have from time to time been adopted, and the fluctuating and uncertain amount of voluntary provision.

#### London's Problem.

On the assumption that there are 30,000 children in London elementary schools for whom some accommodation in a holiday camp or school would be required, the London County Council agreed that 2,000 places should be found in open-air schools in London for tuberculous children, and 2,000 places in open-air schools for the group of children who form the lowest stratum from a health point of view of the remaining 28,000. The next stratum could be provided for in open-air classes in the parks and selected playgrounds. It is not known to what extent these could be developed, but there appears no reason why some 8,000 children should not eventually be accommodated in this way. The younger children may in time be accommodated in nursery schools—say, an additional 4,000. This would leave 14,000 older children whose needs might be met by a period in a holiday or camp school.

The problem is not quite so simple, however, as this, as the range of cases for whom a stay at a holiday or camp school is desirable overlaps the range of cases for whom continuous open-air education is required. Thus, a child may frequently be recommended for an open-air school of the playground class with a stay in a holiday school as a preliminary, and others during the course of their life in an open-air school may from time to time require the added stimulus of a residential camp school for short periods.

#### How it is Done.

A camp school at Bushey Park was placed by the King at the disposal of the Council in the summer of 1919; it was actually opened for the reception of the first draft of boys on August 7. It is situated in the midst of Bushey Park, and consists of a large house—Upper Lodge—and hutments, with offices and outbuildings suitable for the accommodation of 300 boys. The latter structures were erected by the Canadian Red Cross during their temporary occupation of the site. The residential educational staff, which consists of a director and six assistants, is housed in Upper Lodge. The medical arrangements are in the hands of the medical officer, who attends daily at the camp, and two whole-time nurses, one of whom is resident. A dentist attends for five sessions each week. There is a well-equipped medical inspection room and dental clinic; a sick bay is provided for the reception of ailing boys, and an isolation ward is provided for infectious cases. The washing arrangements consist of an adequate supply of shower-baths supplied with hot and cold water; these are being supplemented by the installation of six slipper baths. The sanitary arrangements embrace two sets of offices, one being close to the dormitories. The excellent state of health which has obtained among the boys in the camp up to now affords good evidence of the satisfactory nature of their hygienic surroundings. The dietary is ample and varied; in caloric value it approximates very closely to that provided in the Council's industrial schools.

The admissions to the school are drawn from the ranks of those boys who, by reason of existing anæmia, debility, or malnutrition, appear likely to benefit by a stay in the surroundings afforded. The period for which they are sent down is four weeks, but in exceptional cases it is in the discretion of the camp medical officer to recommend an extension of two weeks. No child under the age of ten is admitted; actively tubercular cases are ineligible, as are mentally defective boys, those suffering from persistent enuresis, and those who do not attain a satisfactory standard of personal hygiene.

#### Beneficial Results.

As regards the progress of the children while in residence at the camp details are available concerning 303 boys.

Heights and Weights.

| No. of Boys. | Length of stay. | Average increase in height. | Average increase of weight. |
|---|---|---|---|
| 36 | 21 days | .17 in. | 2.7 lb. |
| 198 | 28 ,, | .26 in. | 3.3 lb. |
| 69 | 42 ,, | .45 in. | 4.2 lb. |

Five boys lost weight, all during August and probably owing to the very hot weather. The bill of health as a whole was a remarkably clean one; and the results set out above leave no room for doubt in Dr. Hamer's mind as to the beneficial effects arising from a sojourn in the camp. The experiment so admirably described by Dr. Hamer, who gives us the first detailed account of an effort of this kind yet brought to our notice, shows the immense possibilities which lie ahead in the work of educating weakly children under the most favourable conditions and restoring them to a normal state of health, and so turning them into useful citizens.

